# Atlanta Medical College—Clinical Instruction

**Published:** 1866-06

**Authors:** 


*Atlanta Medical College—Clinical Instruction.*—The system of Practical Medicine instituted for the present course in the College promises, thus far, eminent success. One hour every day in the week is devoted exclusively to prescriptions and operations, exclusive of two hours on Wednesdays and Saturdays devoted to lectures and surgical operations on special and interesting cases. A Dispensary is established by the institution in which, after examinations and prescriptions have been made in presence of the class, medicines are furnished the poor of the city, who require treatment. In this way great advantages are afforded the student, and a blessing bestowed on the indigent sick, who daily flock to the dispensary. The hour thus occupied is that which immediately precedes the opening of regular lectures for the day. The systematic course pursued not only affords the class practical lessons in diagnosis, etc., but an opportunity of witnessing the daily progress of each case and the effects of the remedies prescribed. Members of the class are selected to record the cases presented, to write the prescriptions, and to combine the preparations prescribed. The learner is thereby given the opportunity not only to see these practical demonstrations, but to assist, himself, in that with which it is so important to become familiar.

